# Cross-border political competition

**DOI:** 10.1371/journal.pone.0297731

**Published:** 2024-05-29

**Authors:** Jose Segovia-Martin, Óscar Rivero

**Affiliations:** 1 School of Collective Intelligence, M6 Polytechnic University, Rabat, Morocco; 2 Complex Systems Institute of Paris Ile-de-France, Centre national de la recherche scientifique, Paris, France; 3 Mathematics Institute, University of Warwick, Warwick, United Kingdom; 4 Departamento de Matemáticas, Universidad de Santiago de Compostela, Santiago, Spain; University of Massachusetts, UNITED STATES

## Abstract

Individuals are increasingly exposed to news and opinion from beyond national borders. This news and opinion are often concentrated in clusters of ideological homophily, such as political parties, factions, or interest groups. But how does exposure to cross-border information affect the diffusion of ideas across national and ideological borders? Here, we develop a non-linear mathematical model for the cross-border spread of two ideologies. First, we describe the standard deterministic model where the populations of each country are assumed to be constant and homogeneously mixed. We solve the system of differential equations numerically by the Runge-Kutta method and show how small changes in the influence of a minority ideology can trigger shifts in the global political equilibrium. Second, we simulate recruitment as a stochastic differential process for each political affiliation and fit model solutions to population growth rates and voting populations in US presidential elections from 1932 to 2020. We also project the dynamics of several possible scenarios from 2020 to the end of the century. We show that cross-border influence plays a fundamental role in determining election outcomes. An increase in foreign support for a national party’s ideas could change the election outcome, independent of domestic recruitment capacity. One key finding of our study suggests that voter turnout in the US will grow at a faster rate than non-voters in the coming decades. This trend is attributed to the enhanced recruitment capabilities of both major parties among non-partisans over time, making political disaffection less prominent. This phenomenon holds true across all simulated scenarios.

## Introduction

Global connectivity allows for ideological influence of political factions across national borders. This presents a major challenge to the stability of national ideological competition. However, existing models of ideology dynamics have focused on the transmission and evolution of political ideas within a single voting population [1–7]. Surprisingly, despite growing evidence of the impact of social media and international news on voters’ policy preferences and partisan policy responses [8–11], political analysts still tend to examine the success or decline of political parties and candidates almost exclusively in terms of national politics [12–14]. It therefore remains a central question in social, behavioural and political science to understand how exposure to cross-border information affects political competition within the country.

Throughout the 20th century, prominent theories of political competition received intense theoretical and empirical attention in economics [15–20]. According to economists, political competition between political factions basically resembles economic competition [21], a process in which the political equilibrium depends on the ability of individuals and groups to promote their own interests [22]. Under this framework, political entities often engage in strategic behavior to maximize their own benefits, much like firms in a market. This strategic behavior involves the allocation of resources to influence policies and gain electoral support. The Stigler model, for instance, posits that regulation is supplied in response to the demands of the political marketplace, where groups with sufficient resources can secure favorable regulations akin to capturing market share [23]. Similarly, Becker’s model of competition among pressure groups suggests that political outcomes are determined by the efficiency with which groups use their resources to lobby and influence decisions [22]. Thus, political competition can be seen as a market process where political success is driven by the ability to effectively use resources, much like economic competition.

More recently, opinion dynamics models have been used to explore the dynamics of competing opinions by taking into account the interactions between agents [24–27]. These models span from simplistic binary systems such as the voter model [24, 28] to more complex multi-dimensional continuous frameworks [29]. They predominantly simulate two primary sources of influence: peer influence, defined as the direct impact of social peers [30–32], and external influence [27, 33], which encompasses broader societal pressures such as of institutions [25] propaganda [34], fashion [35, 36], or the mass media [37–39]. Particularly relevant to the work we present here is the model of opinion dynamics proposed by Dan Braha and M de Aguilar [40], which suggests that social influence and contagion are likely to intensify as societies become increasingly interconnected through the widespread availability of digital communication and transportation networks. This observation reinforces the foundational assumptions of our model, which aims to simulate national political competition in the context of cross-border social contagion, influenced by both domestic and international pressures in a world with increasing access to global information.

Compartmental models used in epidemiology offer an alternative for modelling political competition as a function of system properties (compartments) affecting contagion. One way to study these dynamic social phenomena is by using nonlinear differential equations [41–43]. The simplest case is to study the influence between political parties within a country’s borders. For example, using an epidemiological approach, Romero et al. [1] investigate ideological propagation in a population of voters where individuals are susceptible to third-party ideology. The authors identified the parameters that most influence third-party voters and rewrote the quantitative results in political terms. The model was applied to the case of the Green Party during the 2000 presidential elections. Ralph Nader, the presidential candidate for the Green Party, won 2% of the popular vote, a percentage that many attributed to the defeat of Democratic candidate Al Gore [44]. The purpose of the model is not so much to predict the voting behaviour of third parties as to offer a set of recruitment strategies to parties so that they might grow and spread within a voting population. They do this by explaining how voter recruitment and political opposition efforts should be managed over time.

Romero et al.’s work focused mainly on third party expansion and was therefore limited in explaining the voting behaviour of two major parties in a two-party system. Building on this model, Misra [2] developed a non-linear mathematical model for the spread of two political parties using an epidemiological approach, where the population is assumed to be constant and homogeneously mixed. Equilibrium and stability analyses as well as numerical simulations were carried out. The model elegantly captures the evolution of the dynamical system depending on the recruitment capacity of the parties. According to Misra’s proposed epidemiology, two conclusions stand out when it comes to predicting the fate of a political party: (i) retaining existing members is more important than recruiting new voters and (ii) small shifts in the movement from one party to another could change voting dynamics faster than expected.

Certainly, the mutual influence between majority parties as well as the effect of third parties and independent candidates have a good chance of having a say in American politics in US presidential elections. However, increasing voter turnout, especially in presidential elections, is turning the attention of political scientists to the influence of non-voters. The 2020 presidential election had the highest voter turnout of the 21st century, with 66.8% of citizens 18 years and older voting in the election, according to voting and registration tables released by the US Census Bureau [45]. Crucially, the U.S. presidential election voter turnout as a share of the total U.S. population grew significantly, albeit with ups and downs, from around 30% in 1932 to around 50% in 2020 [46]. Indeed, non-voters, or more generally those who are undecided or without defined political affiliation have long been considered deserving attention from political scientists [47–53], and turned out to be the decisive force in recent elections. For example, according to Belenky & King [3], the 2004 US presidential election proved wrong the conventional wisdom that swing voters (i.e. voters casting their ballots across potentially opposing ideological lines) are those who have the final say in deciding the outcome in a close US presidential election. As US non-voters still constitute around 40% of the US electorate, whereas swing voters account for around 15% of all eligible voters [3, 46] it seems reasonable to think that non-voters who are increasingly becoming politically active have a growing capacity to change the outcome of an election. Belenky & King [3] captured this idea in a non-linear mathematical model that derives the margin of voters for a particular major party candidate from information on the potential voting behaviour of non-voters.

The idea behind all the previous models is that the influence exerted by individuals from different factions or interest groups within the same voting population is crucial in determining the evolution of the system. Voters and non-voters should all be considered influential forces in the dynamics of ideological competition [47–53]. However, in a world of increasing access to political information beyond national borders, are these within country influences the most decisive and/or only relevant pressures in determining the outcome of national elections? How do changes in the influence of foreign political groups affect the domestic political equilibrium?

The model we present here idealises ideologies as fixed and as competing with each other for supporters both within and across borders. Agents in our model can only support one ideology (party or political tendency) at any given moment in time. First, we describe the standard deterministic model assuming constant homogeneous mixed populations without specific spatial structure. Second, we simulate recruitment as a stochastic differential process for each political group and fit the model solutions to population growth rates and voting populations in US presidential elections from 1932 to 2020. Finally, we project the dynamics of a number of scenarios from 2020 to the end of the century.

## Model

Let us consider two countries whose respective homogeneously distributed populations are *N*_1_ and *N*_2_. The population of country 1 *N*_1_ consists of three sets of agents, namely: (i) without ideological or political affiliation *A*_1_, (ii) with ideology or political affiliation *B*, (iii) with ideology or political affiliation *C*. Similarly, the population of country 2 *N*_2_ consists of three sets of agents, namely (i) without ideological or political affiliation *A*_2_, (ii) with ideology or political affiliation *D*, (iii) with ideology or political affiliation *E*. Now, assume that *B* and *D* have the same ideology, and that *C* and *E* also share the same ideology. These two groups form two blocks of competing ideologies (e.g pro and anti-tax, pro and anti-vaccination, pro and anti-immigration, etc…).

In our model of cross-border influence we will allow that each of the blocks is able to recruit supporters (voters) both within and outside its borders, so that for example, *B* will be able to recruit supporters from *A*_1_ and *C* within its borders, but will also be able to exert influence outside its borders by recruiting agents from *A*_2_ and *E* towards *D*. Similarly, *C*, *D*, *E* will be able to recruit supporters for themselves and for their ideological partners beyond their borders.

Now, consider that there is a rate *μ*_1_ at which agents *μ*_1_*N*_1_ enter the system in country 1, and similarly a rate *μ*_2_ at which agents *μ*_2_*N*_2_ enter the system in country 2. This parameter can be thought of as the rate at which individuals reach the legal age or at which they attain the necessary civic knowledge, skills, cognitive ability and right to vote.

On the other hand, consider there are rates $\mu_{A_{1}}$ and $\mu_{A_{2}}$ at which agents $\mu_{A_{1}}A_{1}$ and $\mu_{A_{2}}A_{2}$ cease to be potential voters of countries 1 and 2 respectively due to death or migration. Similarly, we have death ratios *μ*_*B*_, *μ*_*C*_, *μ*_*D*_ and *μ*_*E*_ for each of the political groups, all labelled with their respective sub-indexes. A system with no gains or losses of citizens over time will keep *μ* constant and of equal value across all population groups.

The parameters *k*_*B*_ and *p*_*B*_ respectively represent the contact ratio and the persuasion probability of group *B* influencing members of group *A*_1_. Specifically, *k*_*B*_ stands for the fraction of *A*_1_ members that are contacted by *B*, while *p*_*B*_ is the likelihood that a contact by *B* results in convincing the contacted member of *A*_1_ to join *B*. Consequently, the term $k_{B}p_{B}A_{1}\frac{B}{N_{1}}$ denotes the rate at which agents transition from *A*_1_ to *B* within country 1, where $\frac{B}{N_{1}}$ reflects the probability of encountering *B* members in the population (i.e. the relative weight of *B* in the population).

Similarly, $k_{C}p_{C}A_{1}\frac{C}{N_{1}}$ stands for the transition rate from *A*_1_ to *C*, $k_{D}p_{D}A_{2}\frac{D}{N_{2}}$ is the rate from *A*_2_ to *D*, and $k_{E}p_{E}A_{2}\frac{E}{N_{2}}$ from *A*_2_ to *E*. Furthermore, agents in *A*_1_ may switch to *B*, influenced not directly by *B* but by *D*, a foreign entity akin to *B*. The transfer of agents from *A*_1_ to *B* due to the influence of *D* occurs at rate $\left(1-k_{B}p_{B}\right)k_{D}p_{D}A_{1}\frac{D}{N_{2}}$. Likewise, *E* captures agents from *A*_1_ to *C* at rate $\left(1-k_{C}p_{C}\right)k_{E}p_{E}A_{1}\frac{E}{N_{2}}$. In country 2, analogous interactions occur between groups *D* and *E*, reflecting a mirrored dynamic of influences akin to those in country 1.

But ideological or political affiliation can fade over time. In the model this loss can be described by a leakage of agents from *B* back to *A*_1_ at rate *γ*_*B*_*B*, and from *C* to *A*_1_ at rate *γ*_*C*_*C*. In country 2, we have the same, there is a leakage of agents from *D* to *A*_2_ at rate *γ*_*D*_*D* and from *E* to *A*_2_ at rate *γ*_*E*_*E*.

Finally, let *ϕ*_*B*_ and *ϕ*_*C*_ be the per capita recruitment capacity of *B* from *C* and of *C* from *B* respectively. Similarly, *ϕ*_*D*_ and *ϕ*_*E*_ are the per capita recruitment capacity of *D* from *E* and of *E* from *D* respectively. Therefore, agents of *C* decide to go to *B* due to *B*’s influence at rate $\phi_{B}C\frac{B}{N_{1}}$ and due to *D*’s influence at rate $\left(1-\phi_{B}\right)\phi_{D}C\frac{D}{N_{2}}$, while agents of *B* decide to go to *C* due to *C*’s influence at rate $\phi_{C}B\frac{C}{N_{1}}$ and due to *E*’s influence at rate $\left(1-\phi_{C}\right)\phi_{E}B\frac{E}{N_{2}}$. Likewise, in country 2 agents of *E* move to *D* due to *D*’s influence at rate $\phi_{D}E\frac{D}{N_{2}}$ and due to *B*’s influence at rate $\left(1-\phi_{D}\right)\phi_{B}E\frac{B}{N_{1}}$, while agents of *D* decide to go to *E* due to *E*’s influence at rate $\phi_{E}D\frac{E}{N_{2}}$ and due to *C*’s influence at rate $\left(1-\phi_{E}\right)\phi_{C}D\frac{C}{N_{1}}$.

This modeling framework facilitates understanding the dynamics of population movements influenced by both within and between-country interactions.

In accordance with the parameters, terms and assumptions described above, the governing differential equations of the model can be written as follows:

$$\begin{array}{c} \left\{ \begin{array}{cc} \frac{dA_{1}}{dt}= & \mu_{1}N_{1}-k_{B}p_{B}A_{1}\frac{B}{N_{1}}-\left(1-k_{B}p_{B}\right)k_{D}p_{D}A_{1}\frac{D}{N_{2}}-k_{C}p_{C}A_{1}\frac{C}{N_{1}}-\left(1-k_{C}p_{C}\right)k_{E}p_{E}A_{1}\frac{E}{N_{2}}\\ & -\mu_{A_{1}}A_{1}+\gamma_{B}B+\gamma_{C}C\\ \frac{dB}{dt}= & k_{B}p_{B}A_{1}\frac{B}{N_{1}}+\left(1-k_{B}p_{B}\right)k_{D}p_{D}A_{1}\frac{D}{N_{2}}-\phi_{C}B\frac{C}{N_{1}}-\left(1-\phi_{C}\right)\phi_{E}B\frac{E}{N_{2}}+\phi_{B}C\frac{B}{N_{1}}\\ & +\left(1-\phi_{B}\right)\phi_{D}C\frac{D}{N_{2}}-\mu_{B}B-\gamma_{B}B\\ \frac{dC}{dt}= & k_{C}p_{C}A_{1}\frac{C}{N_{1}}+\left(1-k_{C}p_{C}\right)k_{E}p_{E}A_{1}\frac{E}{N_{2}}-\phi_{B}C\frac{B}{N_{1}}-\left(1-\phi_{B}\right)\phi_{D}C\frac{D}{N_{2}}+\phi_{C}B\frac{C}{N_{1}}\\ & +\left(1-\phi_{C}\right)\phi_{E}B\frac{E}{N_{2}}-\mu_{C}C-\gamma_{C}C\\ \frac{dA_{2}}{dt}= & \mu_{2}N_{2}-k_{D}p_{D}A_{2}\frac{D}{N_{2}}-\left(1-k_{D}p_{D}\right)k_{B}p_{B}A_{2}\frac{B}{N_{1}}-k_{E}p_{E}A_{2}\frac{E}{N_{2}}-\left(1-k_{E}p_{E}\right)k_{C}p_{C}A_{2}\frac{C}{N_{1}}\\ & -\mu_{A_{2}}A_{2}+\gamma_{D}D+\gamma_{E}E\\ \frac{dD}{dt}= & k_{D}p_{D}A_{2}\frac{D}{N_{2}}+\left(1-k_{D}p_{D}\right)k_{B}p_{B}A_{2}\frac{B}{N_{1}}-\phi_{E}D\frac{E}{N_{2}}-\left(1-\phi_{E}\right)\phi_{C}D\frac{C}{N_{1}}+\phi_{D}E\frac{D}{N_{2}}\\ & +\left(1-\phi_{D}\right)\phi_{B}E\frac{B}{N_{1}}-\mu_{D}D-\gamma_{D}D\\ \frac{dE}{dt}= & k_{E}p_{E}A_{2}\frac{E}{N_{2}}+\left(1-k_{E}p_{E}\right)k_{C}p_{C}A_{2}\frac{C}{N_{1}}-\phi_{D}E\frac{D}{N_{2}}-\left(1-\phi_{D}\right)\phi_{B}E\frac{B}{N_{1}}+\phi_{E}D\frac{E}{N_{2}}\\ & +\left(1-\phi_{E}\right)\phi_{C}D\frac{C}{N_{1}}-\mu_{E}E-\gamma_{E}E \end{array}\right. \end{array}$$ (1)

**Fig 1:**
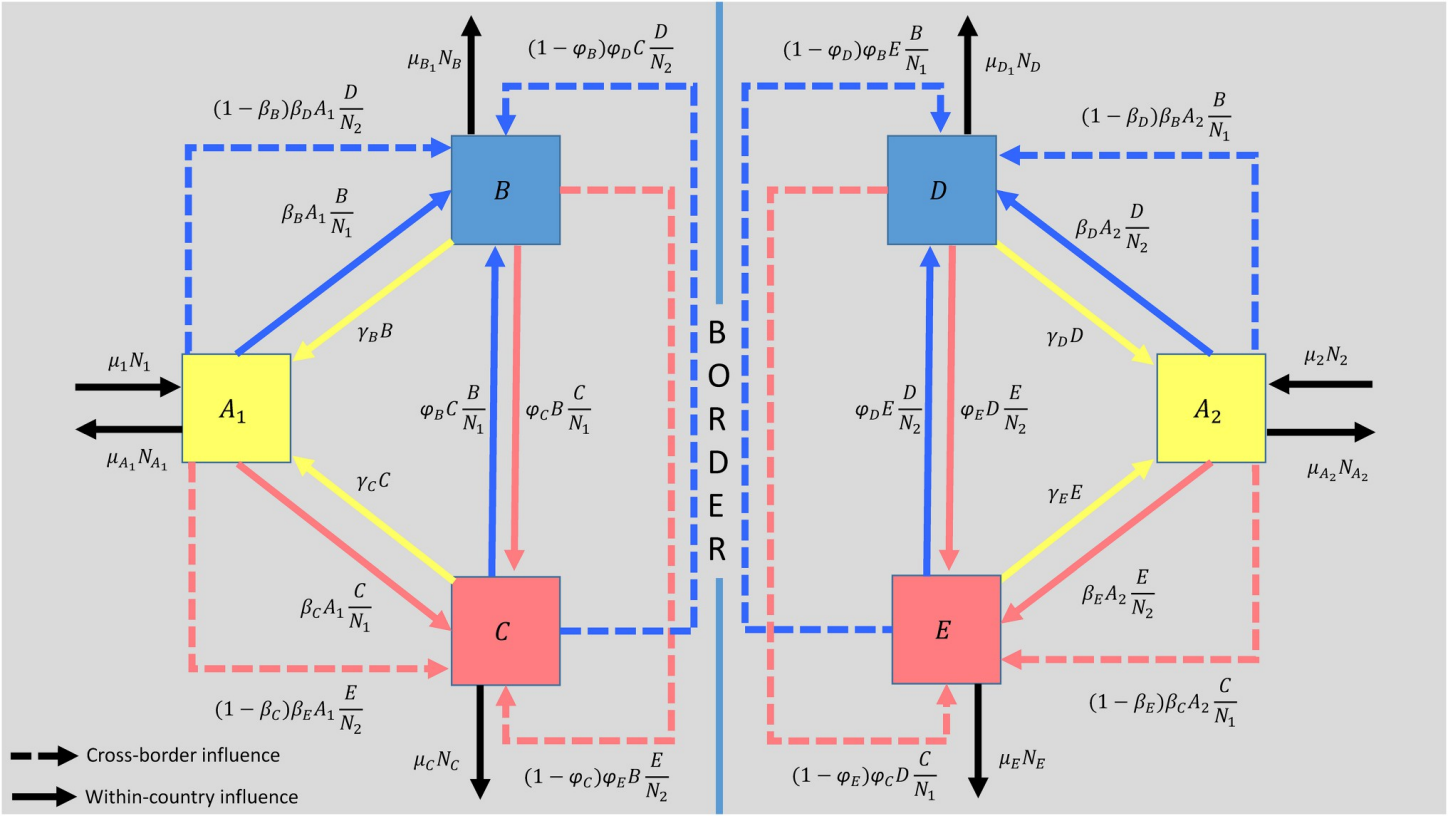
Ilustrative figure of the dynamical system.

Fig 1 illustrates the dynamical system, with the inflows and outflows of individuals in each political group within each country. *N*_1_, *A*_1_, *B*, *C*, *N*_2_, *A*_2_, *D*, *E* are all dependent on time. For brevity of notation, the time dependencies of *N*_1_(*t*), *A*_1_(*t*), *B*(*t*), *C*(*t*), *N*_2_(*t*), *A*_2_(*t*), *D*(*t*), *E*(*t*) are not made explicit in the equations throughout the paper.

This system can be simplified, as we show in Appendix A, to a reduced system containing four differential equations.

$$\begin{array}{c} \left\{ \begin{array}{c} \frac{db}{dt}=\beta_{B}\left(1-b-c\right)b+\left(1-\beta_{B}\right)\beta_{D}\left(1-b-c\right)d-\phi_{w_{1}}bc-\phi_{b_{1}}be-\mu_{B}b-\gamma_{B}b\\ \frac{dc}{dt}=\beta_{C}\left(1-b-c\right)c+\left(1-\beta_{C}\right)\beta_{E}\left(1-b-c\right)e+\phi_{w_{1}}bc+\phi_{b_{1}}be-\mu_{C}c-\gamma_{C}c\\ \frac{dd}{dt}=\beta_{D}\left(1-d-e\right)d+\left(1-\beta_{D}\right)\beta_{B}\left(1-d-e\right)b-\phi_{w_{2}}de-\phi_{b_{2}}dc-\mu_{D}d-\gamma_{D}d\\ \frac{de}{dt}=\beta_{E}\left(1-d-e\right)e+\left(1-\beta_{E}\right)\beta_{C}\left(1-d-e\right)c+\phi_{w_{2}}de+\phi_{b_{2}}dc-\mu_{E}e-\gamma_{E}e \end{array}\right. \end{array}$$ (2)

This system of equations may be understood as follows: *b* (resp. *c*) represents the proportion of agents with political affiliation *B* (resp. *C*) in the first country, while *d* (resp. *e*) represents the proportion of agents with political affiliation *D* (resp. *E*) in the second country. In particular, 1 − *b* − *c* (resp. 1−*d* − *e*) represents the proportion of citizens with no political affiliation in the first country (resp. in the second country). Then, the variation of these four proportions are dictated by the aforementioned parameters, which are of three kinds: the *β*_*i*_ have to do with the potentiality to attract new unaffiliated voters; the *ϕ*_*i*_ are related with the changes and transfers among the different parties; and the *μ*_*i*_ have to do with the decay of supporters of a certain option.

## Numerical simulations

### A political butterfly effect

We conducted numerical simulations assuming constant *μ* = 0.016 and constant *γ* = 0.01. We assume that the average individual acquires the right to vote at the age of 18 and spends about 62.5 years of his or her life with an active political life, i.e. *μ* = 1/62.5 = 0.016. We systematically manipulated the recruitment capacity parameters *β* and *ϕ*, which measure a political group’s effectiveness in recruiting from non-partisans and from members of other political groups, respectively. Following the logic of previous models, we keep these parameters at realistically low values ranging from 0 to 0.2. For instance, a value of *β* = 0.04 means that it takes 25 members of a political party to recruit one voter within a year from the non-partisan group. Similarly, a value of *ϕ* = 0.04 indicates that it takes 25 members of a political party to recruit one voter within a year from another political group.

**Fig 2:**
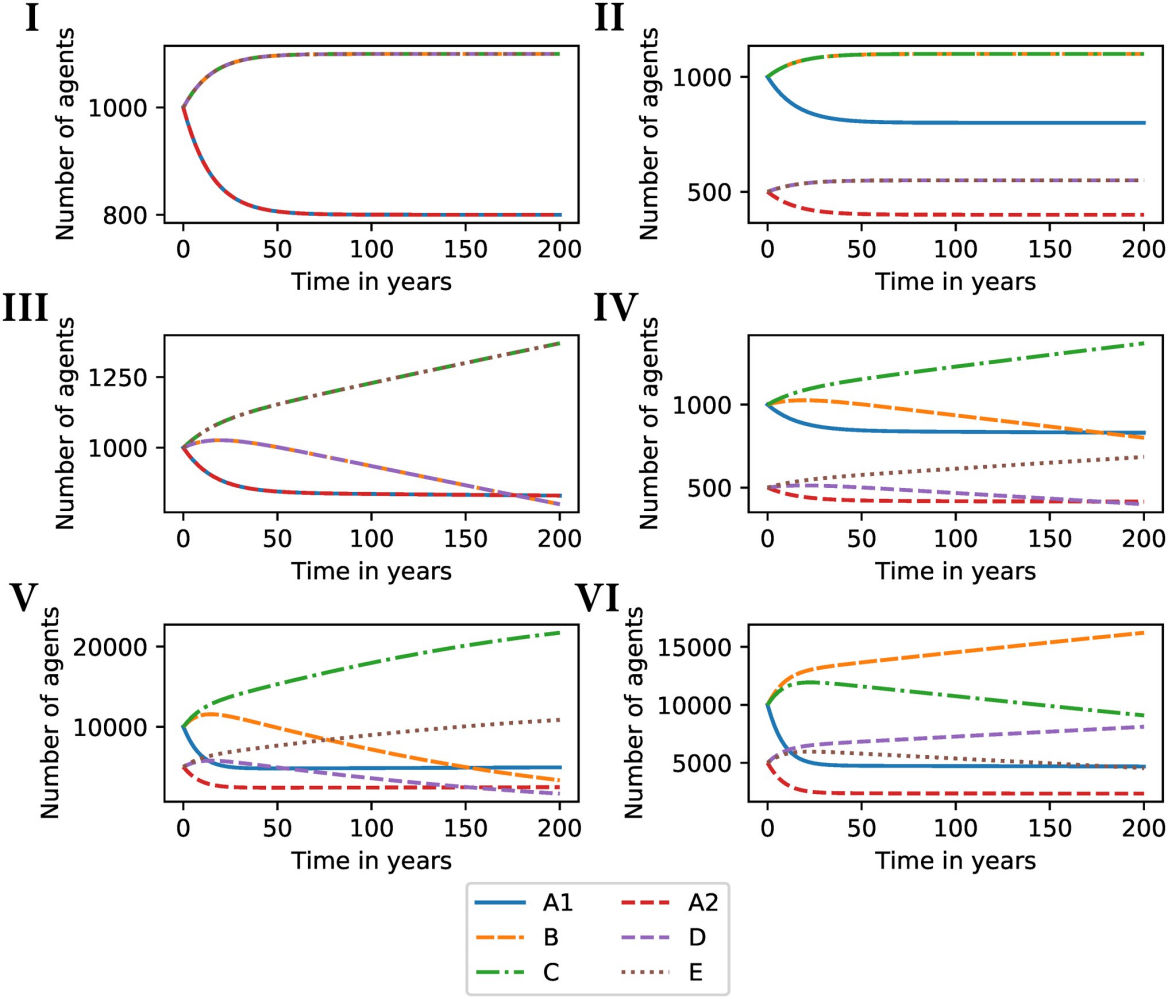
Number of agents supporting each ideological bloc over time. I: Simulations for same initial population size (*A*_1_ = *B* = *C* = *A*_2_ = *D* = *E*) and same influence, with parameters *β*_*B*_ = *β*_*C*_ = *β*_*D*_ = *β*_*E*_ = 0.05 and *ϕ*_*B*_ = *ϕ*_*C*_ = *ϕ*_*D*_ = *ϕ*_*E*_ = 0.02. II: Simulations for different initial population size (*A*_1_/2 = *B*/2 = *C*/2 = *A*_2_ = *D* = *E*) and same influence, with parameters *β*_*B*_ = *β*_*C*_ = *β*_*D*_ = *β*_*E*_ = 0.05 and *ϕ*_*B*_ = *ϕ*_*C*_ = *ϕ*_*D*_ = *ϕ*_*E*_ = 0.02. III: Simulations for same initial population size (*A*_1_ = *B* = *C* = *A*_2_ = *D* = *E*) and different influence, with *β*_*B*_ = 0.04 and parameters *β*_*C*_ = *β*_*D*_ = *β*_*E*_ = 0.05 and *ϕ*_*B*_ = *ϕ*_*C*_ = *ϕ*_*D*_ = *ϕ*_*E*_ = 0.02. IV: Simulations for different initial population size (*A*_1_/2 = *B*/2 = *C*/2 = *A*_2_ = *D* = *E*) and different influence, with *β*_*B*_ = 0.04 and parameters *β*_*C*_ = *β*_*D*_ = *β*_*E*_ = 0.05 and *ϕ*_*B*_ = *ϕ*_*C*_ = *ϕ*_*D*_ = *ϕ*_*E*_ = 0.02. V and VI: Simulations for different initial population size (*A*_1_/2 = *B*/2 = *C*/2 = *A*_2_ = *D* = *E*) and different influence, with *β*_*B*_ = 0.12, *β*_*C*_ = 0.04, *β*_*D*_ = 0.06, *β*_*E*_ = 0.12 and *ϕ*_*B*_ = 0.01, *ϕ*_*C*_ = 0.03, *ϕ*_*E*_ = 0.01. With these parameters, there is an inflection point around *ϕ*_*D*_ = 0.0267 whose values above and below determine the domains of the success function of one or the other competing ideology.

**Fig 3:**
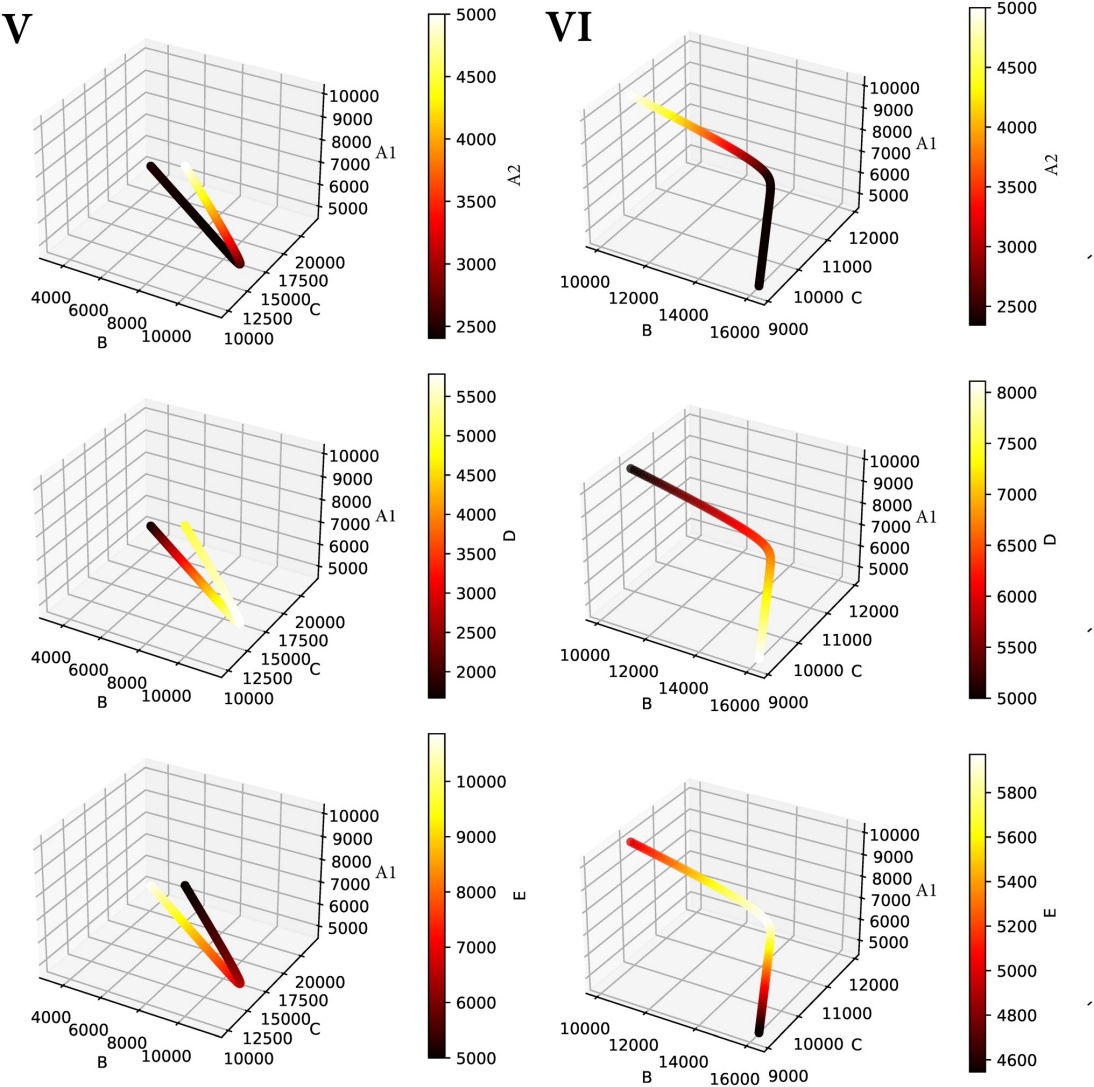
Phase portraits of scenarios V (*ϕ*_*D*_ = 0.02) and VI (*ϕ*_*D*_ = 0.03). Representation of the trajectories of solutions of the dynamical system in the phase plane for the initial conditions in V and VI. Parameters: *A*_1_/2 = *B*/2 = *C*/2 = *A*_2_ = *D* = *E*, and *k*_*B*_ = 0.6, *k*_*C*_ = 0.4, *k*_*D*_ = 0.6, *k*_*E*_ = 0.6, *p*_*B*_ = 0.2, *p*_*C*_ = 0.1, *p*_*D*_ = 0.1, *p*_*E*_ = 0.2, *ϕ*_*B*_ = 0.01, *ϕ*_*C*_ = 0.03, *ϕ*_*E*_ = 0.01.

Our simulations show various equilibria, some with co-dominance of ideologies (Fig 2I and 2II), and others with dominance of one ideology (Fig 2III and 2IV). Interestingly, in some areas of the parameter space, a small change in the recruiting capacity of one of the parties can produce a total reversal of the balance of power. This butterfly effect is illustrated by the comparison of scenarios V and VI in Fig 2, which makes it clear that the system suffers from deterministic chaos. An increase as small as 0.005 in the initial recruitment capacity of the minority party *D* from *E* in country *N*_2_ triggers a global ideological shift, revealing a high sensitivity of the system to initial conditions. For illustrative purposes, we implemented arbitrarily small changes of 0.5% in the examined parameters. Any smaller change delays the effects, but all other conclusions hold. According to our model, these seemingly imperceptible shifts in influence around the tipping point can consummate short-term political changes (within a few decades) and extinctions of once-dominant ideologies within a few hundred years. For illustrative purposes, Fig 3 shows the geometric representations of the dynamical system in the 4-dimensional phase space of *N*_1_ with each ideology of *N*_2_, for the numerical simulations of scenarios V and VI.

## Equilibrium analysis

In this part, we develop a standard fixed-point analysis to discuss, from a mathematical perspective, some of the equilibrium points of the system. Further, we interpret them using the approach of the article, discussing their significance and consequences. Note that the different equilibrium points are obtained by imposing the condition

$$\begin{array}{c} \frac{db}{dt}=\frac{dc}{dt}=\frac{dd}{dt}=\frac{de}{dt}=0. \end{array}$$

This gives the four equations

$$\begin{array}{cc} \beta_{B}\left(1-b-c\right)b+\left(1-\beta_{B}\right)\beta_{D}\left(1-b-c\right)d-\phi_{w_{1}}bc-\phi_{b_{1}}be-\mu_{B}b-\gamma_{B}b & =0\\ \beta_{C}\left(1-b-c\right)c+\left(1-\beta_{C}\right)\beta_{E}\left(1-b-c\right)e+\phi_{w_{1}}bc+\phi_{b_{1}}be-\mu_{C}c-\gamma_{C}c & =0\\ \beta_{D}\left(1-d-e\right)d+\left(1-\beta_{D}\right)\beta_{B}\left(1-d-e\right)b-\phi_{w_{2}}de-\phi_{b_{2}}dc-\mu_{D}d-\gamma_{D}d & =0\\ \beta_{E}\left(1-d-e\right)e+\left(1-\beta_{E}\right)\beta_{C}\left(1-d-e\right)c+\phi_{w_{2}}de-\phi_{b_{2}}dc-\mu_{E}e-\gamma_{E}e & =0. \end{array}$$

This is a system of four second degree equations, so we may expect the existence of different solutions (16 for a general choice of coefficients, although several of them may not satisfy the constraints on the variables, like *b*, *c*, *d*, *e* ∈ [0, 1]), and moreover in some degeneracy cases that we will explore later on it may happen that there are infinitely many. We would like to highlight some different cases of special relevance in our subsequent analysis.

### An apolitical scenario

Observe that *b* = *c* = *d* = *e* = 0 is always a solution for the previous system, corresponding to a situation where all the citizens present non political affiliation or ideology. This would be the case, for example, when the coefficients *γ*_*i*_ are quite large and there is a substantial leakage in the support to the different political options. This is also the case when the coefficients *μ*_*B*_, *μ*_*C*_, *μ*_*D*_, *μ*_*E*_ are large (population decay), since their role is symmetric with respect to the coefficients *γ*_*i*_.

This situation is not observed in the different experiments we have performed, since it is highly unlikely according to the values of the parameters we can expect from a realistic model. In concrete, this corresponds to a situation where the citizens are not involved at all in politics (predemocratic scenarios).

### Coexistence of both political options

In the cases where all the *β*_*i*_*ϕ*_*i*_, *μ*_*i*_, and *γ*_*i*_ agree, we can find explicit solutions satisfying the condition *b* = *c* = *d* = *e*. In this scenario, the system degenerates to a single equation, and the common value of *b* = *c* = *d* = *e* is given by the equation

$$\begin{array}{c} b(\beta (1-2b)+\beta (1-\beta )(1-2b)-\mu -\gamma )=0, \end{array}$$

whose solutions are given by *b* = *c* = *d* = *e* = 0 (already discussed before) and

$$\begin{array}{c} b=c=d=e=\frac{1-\frac{\mu }{2\beta -\beta^{2}}}{2}. \end{array}$$

This is the case which arises in our simulations in scenarios I and II, in which this common value is around *b* = *c* = *d* = *e* ≈ 0.366. This gives a situation (scenario I) in which the representatives of each option are 1100 in each population, and those who show an apolitical behaviour are 800 (values approximated to the closest integer). In the second case, in which the size of the second population is different, the analysis works verbatim, with the unique difference that the number of representatives for each option in the second population will be half of the others (550, 550 and 400, respectively). It is relevant to observe that the size of the population is not relevant for our analysis, since only proportions matter.

This situation is indeed rather plausible: the coexistence of both options is stable and both of them have a fixed proportion of supporters. This would be the case, for example, when there are not many interactions between the different political options and people are not likely to modify their views. It can be considered a representation of what happens in many European or American countries nowadays.

### Complete domain of a political option

Consider the situation where *b* = *d* = 0. Both the first and third equations become 0 = 0, so we are left with a system of two equations:

$$\begin{array}{cc} \beta_{C}\left(1-c\right)c+\left(1-\beta_{C}\right)\beta_{E}\left(1-c\right)e-\mu_{C}c-\gamma_{C}c & =0\\ \beta_{E}\left(1-e\right)e+\left(1-\beta_{E}\right)\beta_{C}\left(1-e\right)c-\mu_{E}e-\gamma_{E}e & =0. \end{array}$$

This is a system of two second degree equations where we can do a more explicit analysis. First of all, and to get an intuition of what is going on, consider the symmetric case, in which *β*: = *β*_*C*_ = *β*_*E*_, *μ*: = *μ*_*C*_ = *μ*_*E*_ and *γ*: = *γ*_*C*_ = *γ*_*E*_ and look for the solutions in which *c* = *e* ≠ 0. This yields

$$\begin{array}{c} c=e=1-\frac{\mu +\gamma }{\beta (2-\beta )}. \end{array}$$

Note however that we do not need the symmetry condition. For a general choice of parameters, as shown below, this gives a unique non-trivial solution. As in the first case, this situation corresponds to a case where one political party has disappeared, but now the other has some impact (while the rest of the population is apolitical).

Finally, observe we also get solutions putting *c* = *e* = 0. In this case, we have the system

$$\begin{array}{cc} \beta_{B}\left(1-b\right)b+\left(1-\beta_{B}\right)\beta_{D}\left(1-b\right)d-\mu_{B}b-\gamma_{B}b & =0\\ \beta_{D}\left(1-d\right)d+\left(1-\beta_{D}\right)\beta_{B}\left(1-d\right)b-\mu_{D}d-\gamma_{D}d & =0. \end{array}$$

For example, in the scenario III (and IV) this yields the system

$$\begin{array}{cc} 0.04(1-b)b+0.96\cdot 0.05(1-b)d-0.026b & =0\\ 0.05(1-d)d+0.95\cdot 0.04(1-d)b-0.026d & =0, \end{array}$$

whose solutions are *b* = *c* = *d* = *e* = 0 and *c* = *e* = 0, *b* = *d* ≈ 0.705. Hence, in this situation, the presence of the unique remaining option is stronger than in the previous cases. This is coherent with the result we obtain: the dominant value will converge to a value of 2114; the non-dominant one will tend to disappear; and the number of apolitical citizens will stabilize around 886. In the case of scenario IV the situation will be analogous, since in this case only the relative proportions with respect to each population matter.

The case shown in scenarios V and VI can be analyzed in this same framework: the former corresponds to the case in which *c* = *e* = 0, which gives the solution *b* = *d* ≈ 0.850 (around 25486 and 12743 supporters for the dominant option in the two populations); the latter represents the solution *b* = *d* = 0, with *c* = *e* ≈ 0.850 (around 25486 and 12743 people in the dominant groups).

This shows that for certain choices of parameters, one of the options can completely dominate the political scene. It represents a plausible setting in which a political option has decayed a lot and a certain political ideology has taken the lead without any relevant opposition (societies which are not very polarized and have a solid leadership). This is the case in the so-called dominant-party system, a political occurrence in which a single political party continuously dominates election results over running opposition groups or parties. According to [54], between 1950 and 2017, more than 130 countries were included in the list of dominant-party systems at different times.

### More complex situations

Until now, we have discussed situations in which either some of the variables was 0 (that is, one of the political options disappeared after some time) or all the proportions were the same. The system of equations we have considered is however more sophisticated, and there could be solutions where all four values different from zero, although it is not feasible to give analytic expressions for the solutions in the more general situation. We discuss two of these situations that we believe can be of potential interest.

(a) Consider a symmetric situation where *β*_*B*_ = *β*_*D*_, *β*_*C*_ = *β*_*E*_, $\phi_{w_{1}}=\phi_{w_{2}}$, $\phi_{b_{1}}=\phi_{b_{2}}$, and all the *μ*_*i*_ and *γ*_*i*_ equal (call them *μ* and *γ*, respectively). Then, we can look for non-zero solutions of the form *b* = *d* and *c* = *e*. We thus obtain the system
  $$\begin{array}{cc} \left(\beta_{B}+\beta_{D}-\beta_{B}\beta_{D}\right)\left(1-b-c\right)-\left(\phi_{w_{1}}+\phi_{b_{1}}\right)c-\left(\mu +\gamma \right) & =0\\ \left(\beta_{B}+\beta_{D}-\beta_{B}\beta_{D}\right)\left(1-b-c\right)c+\left(\phi_{w_{1}}+\phi_{b_{1}}\right)b-\left(\mu +\gamma \right) & =0 \end{array}$$
  This is a linear system, which is compatible and has a unique (and explicit) solution provided that $\phi_{w_{1}}+\phi_{b_{1}}\neq 0$. From a political perspective, this represents a situation in which the two parties have the same proportion of supporters in both populations, but neither the major option is able to make a change on the minority nor the other way around.
(b) Consider now a situation where *β*_*B*_ = *β*_*E*_, *β*_*C*_ = *β*_*D*_, $\phi_{w_{1}}=-\phi_{w_{2}}$, $\phi_{b_{1}}=-\phi_{b_{2}}$, and all the *μ*_*i*_ and *γ*_*i*_ are equal. We can look for solutions where *b* = *e* and *c* = *d*, and then, the equation ostensibly simplify since two of them are redundant. We thus obtain the system
  $$\begin{array}{cc} \left(\beta_{B}+\left(1-\beta_{B}\right)\beta_{D}\right)\left(1-b-c\right)-\left(\phi_{w_{1}}+\phi_{b_{1}}\right)c-\left(\mu +\gamma \right) & =0\\ \left(\beta_{C}+\left(1-\beta_{C}\right)\beta_{E}\right)\left(1-b-c\right)+\left(\phi_{w_{1}}-\phi_{b_{1}}\right)b-\left(\mu +\gamma \right) & =0 \end{array}$$
  The system is compatible and has a unique solution with *b* = *e* and *c* = *d*, unless $\phi_{w_{1}}+\phi_{b_{1}}=0$. In the precise case in which *β*_*B*_ + (1 − *β*_*B*_)*β*_*D*_ = *β*_*C*_ + (1− *β*_*C*_)*β*_*E*_, the system has infinitely many solutions. Indeed, for that choice of parameters we get that any option with
  $$\begin{array}{c} b+c=1-\frac{\mu +\gamma }{\beta_{B}+\left(1-\beta_{B}\right)\beta_{D}} \end{array}$$
  works (and *d* + *e* having the same value). This means that the equilibrium point is characterized by a prescribed proportion of apolitical citizens in both countries, but any other distribution among blocks can be an equilibrium point.
  This represents a different and interesting case, in which the dominant party is different in both regions but the exchanges among them do not cause a change. We may change about that as a phenomenon occurring between two closely geographic regions, with important economical differences leading to a distinct hegemonic party on each of them.
(c) Note that all the situations we have analyzed from an analytical point of view are based on certain symmetries between the coefficients. For a general choice of coefficients we should expect less straightforward solutions, where a priori one could find non-symmetric solutions where both political options have different (and non-zero) values in each population. For a random choice of parameters, we do not expect any general solution for the system of equations, and one must rely on the usual numerical methods to get the equilibrium points.

## Stability analysis

To discuss the stability of the system, we use the standard linearization procedure, by studying the matrix of derivatives at the equilibrium points. Depending on whether or not that matrix is negative definite, we will have stable equilibrium or not. As in the previous section, we will illustrate a few relevant cases, similar to the ones arising in our simulations, since the treatment of a general setting is not feasible by analytic means and one needs to resort to numerical tests.

### An apolitical scenario

Let us begin by dealing with the solution (0, 0, 0, 0). The matrix of derivatives in that case is

$$\left(\begin{array}{cccc} \beta_{B}-\mu_{B}-\gamma_{B} & 0 & \left(1-\beta_{B}\right)\beta_{D} & 0\\ 0 & \beta_{C}-\mu_{C}-\gamma_{C} & 0 & \left(1-\beta_{C}\right)\beta_{E}\\ \left(1-\beta_{D}\right)\beta_{B} & 0 & \beta_{D}-\mu_{D}-\gamma_{D} & 0\\ 0 & \left(1-\beta_{E}\right)\beta_{C} & 0 & \beta_{E}-\mu_{E}-\gamma_{E} \end{array}\right)$$

Depending on the values of the parameters, this could be an equilibrium point or not. Let us discuss the two limit cases to illustrate this phenomenon.

- If *β*_*B*_ = *β*_*C*_ = *β*_*D*_ = *β*_*E*_ = 0, the equilibrium is stable. This means that since the only inputs are related to the increase of political disaffection, there is an exponential decay to a situation in which everyone is apolitical.
- If all the *μ*_*i*_ and *γ*_*i*_ are equal to zero, the eigenvalues of the matrix are *β*_*B*_*β*_*D*_, *β*_*C*_*β*_*E*_, 1 − (1 −*β*_*B*_)(1 − *β*_*D*_) and 1 − (1 − *β*_*C*_)(1 − *β*_*E*_). All of them are positive, provided that the *β*_*i*_ are strictly between 0 and 1, so the equilibrium is unstable.

More generally, the stability will depend on the size of the parameters *β*_*i*_ with respect to the *μ*_*i*_ and *γ*_*i*_. When the latter are dominant, the system is stable at (0, 0, 0, 0); elsewhere, it will be an unstable point. This is coherent with our expectation that a scenario like that is not expected in a *normal modern* society.

### Coexistence of both political options

Let us consider a case where all the *β*_*i*_*ϕ*_*i*_, *μ*_*i*_, and *γ*_*i*_ agree, and further the four variables *b*, *c*, *d* and *e* agree at the initial condition. Then, we can restrict to symmetric solution in which not only the equilibrium corresponds to *b* = *c* = *d* = *e*, but in the evolution of the system all four variables are equal all the time (as it happens in scenarios I and II, and which would correspond to two more or less equally strong blocks with similar influence).

In this situation, the condition for stability is that

$$\begin{array}{c} \left(2\beta -\beta^{2}\right)\left(1-4b\right)-\mu =2\left(2\beta -\beta^{2}-\mu \right)-\mu <0. \end{array}$$

In the case analyzed in scenarios I and II these hypotheses clearly holds. This is consistent with the fact that a scenario where both political options coexist with a (more or less) stable number of supporters is frequent in a great number of modern societies.

### Complete domain of a political option

We now discuss the stability at point which *b* = *d* and *c* = *e* = 0, as those arising in most of the scenarios displayed in the examples (cases III to VI, if we include the symmetric situation of *c* = *e* and *b* = *d* = 0). Although this can be done in full generality, let us restrict to the case in which the initial conditions agree and we have *b* = *d* and *c* = *e* since the initial moment. As it happened before, the character of the matrix depends on the different coefficients involved: if the *ϕ*_*wi*_, *ϕ*_*bi*_, *μ*_*i*_ and *γ*_*i*_ are zero, the matrix will not be negative definite, while in a situation where all the *β*_*i*_ are zero, we have the opposite phenomenon. More precisely, the system is governed by two equations:

$$\begin{array}{cc} \beta_{B}\left(1-b-c\right)b+\left(1-\beta_{B}\right)\beta_{D}\left(1-b-c\right)d-\phi_{w_{1}}bc-\phi_{b_{1}}be-\mu_{B}b-\gamma_{B}b & =0\\ \beta_{D}\left(1-d-e\right)d+\left(1-\beta_{D}\right)\beta_{B}\left(1-d-e\right)b-\phi_{w_{2}}de-\phi_{b_{2}}dc-\mu_{D}d-\gamma_{D}d & =0. \end{array}$$

Then, the matrix of derivatives is given by

$$\left(\begin{array}{cc} \left(\beta_{B}+\beta_{D}-\beta_{B}\beta_{D}\right)\left(1-2b\right)-\left(\mu +\gamma \right) & -(\beta_{B}+\beta_{D}-\beta_{B}\beta_{D})b\\ 0 & -\left(\beta_{C}+\beta_{E}-\beta_{C}\beta_{E}\right)\left(1-b\right)-\left(\mu +\gamma \right). \end{array}\right)$$

To discuss a plausible situation, consider the scenario III (and IV), in which *b* = *d* ≈ 0.705. Then, the condition of stability is that the two diagonal entries are negative, which immediately follows if *b* ∈ (1/2, 1).

Let us take a closer look to the situation of scenarios V and VI: both of them represent a stable equilibrium, and indeed the stability does not involve at all the value of the parameters *ϕ*_*i*_. This is indeed a system with (at least) two equilibrium points, and the convergence to one or the other will depend on the initial conditions (and can be represented in terms of a phase portrait). In particular, the evolution of the system and the phase portray are sensible to the values of the *ϕ*_*i*_, and for a fixed initial condition converging to one equilibrium point or the other will depend on all these choices, as the different behaviors of scenarios V and VI show.

### More complex scenarios

This same method can be applied to study other settings, like those in which all the values of *b*, *c*, *d*, *e* are non-zero. In this case, it is not possible to make any kind of general statement, since there are many different parameters and depending on the relation among them we can get stable or unstable equilibrium. As a general philosophy, large values of the coefficients *μ*_*i*_ and *γ*_*i*_ will contribute to stability.

## Testing the model with US data

In this model, *N*_1_ corresponds to the United States and *N*_2_ represents the population outside the US in potential contact with the US. *B* and *C* are Democrats and Republicans, respectively, representing the major US political parties, and *D* and *E* symbolise parties of political movements outside the US that are sympathetic to these US political factions. We collected data on voting populations and the number of votes cast in US presidential elections from 1932 (the first year with publicly available data on the voting-age population) to 2020. We used the US Census Bureau as the primary data source. The popular vote has traditionally been dominated by Democrats in the collected time series. In the 23 presidential elections since 1932, Republicans have won the popular vote only 8 times, compared to 15 for Democrats. The total number of votes cast for Democrats and Republicans has accumulated to approximately 957.39 million and 942.23 million votes, respectively. As the population has grown, the share of the population participating in these elections has gradually increased. Despite this increase, turnout has never reached half of the total population. This is partly due to the proportion of the population under eighteen years of voting age, but also because many potential voters over eighteen do not participate, or are ineligible to vote. We reconstructed the abstention level for each election by subtracting the total votes cast from the eligible voting population, and equated this metric to the *A*_1_ group, i.e., the non-partisan group in country *N*_1_. For agents outside the U.S., it is assumed that political tendencies are evenly split at the start of the simulation, with one-third non-partisan, one-third pro-Democrats, and one-third pro-Republicans. This assumption allows us to explore the dynamics of political influence under equivalent initial conditions, recognizing that actual alignments may differ based on regional specifics not captured in this simplification. The model is versatile and can be adapted to fit initial conditions corresponding to specific cases (e.g., established democracies with comparable two-party systems such as Canada, the UK, and Australia).

### Stochastic model solutions for realistic conditions

We fitted the model solutions to the data and projected the dynamics of a number of possible scenarios from 2020 to the end of the century. We assumed the rate of change in population growth, $\frac{dr}{dt}$, to follow linear functions: $\frac{dr}{dt}=0.018-0.0001t$ for the United States and $\frac{dr}{dt}=0.023-0.0001t$ for the population outside the US, reflecting the historically higher rate of population growth outside the US over the last 80 years. Considering the stochastic nature of recruitment processes, we modeled each political affiliation using the stochastic differential equations *dx* = *g*(*t*, *x*)*dt* + *h*(*t*, *x*)*dW*, where *g*(*t*, *x*) represents the deterministic component of change, and *h*(*t*, *x*) represents the stochastic component. We discretised the Wiener process at each time step *dt* as $dW∼\sqrt{dt}N\left(0,1\right)$, where *N*(0, 1) is a normal distribution with mean 0 and variance 1.

The model that best fits the actual data is a model with *k*_*B*_ = *k*_*C*_ = 0.55, *k*_*D*_ = *k*_*E*_ = 0.1, *p*_*B*_ = *p*_*C*_ = 0.15, *p*_*D*_ = *p*_*E*_ = 0.1. Given that *k*_*B*_*p*_*B*_ and *k*_*C*_*p*_*C*_, determine the per capita recruitment rates of Democrats *B* and Republicans *C*, respectively, from the non-partisan group *A*_1_, we have that the best-fit model for the United States is a model where the average per capita political influence of the US is $\frac{\beta_{B}+\beta_{C}}{\beta_{D}+\beta_{E}}=8.25$ times greater than the per capita influence of the aligned political factions outside the US. Within this region of parameter space, we systematically manipulated the initialisation values of the system according to Tables 1 and 2.

**Table 1:** Value parameter combinations examined.

	Initial value parameter combinations (*t*_0_ = 1932)		
Scenarios from 2020	*ϕ*_*B*_ = *ϕ*_*C*_ = 0.05 and 1932 electoral results	*ϕ*_*B*_ = 0.05, *ϕ*_*C*_ = 0.055 and 1932 electoral results	*ϕ*_*B*_ = *ϕ*_*C*_ = 0.05 and electoral tie
*ϕ*_*D*_ = *ϕ*_*E*_ = 0.01, *γ*_*B*_ = *γ*_*C*_ = *γ*_*D*_ = *γ*_*E*_ = 0.01	S0000	S0100	S1000
*ϕ*_*D*_ = 0.015, *ϕ*_*E*_ = 0.01, *γ*_*B*_ = *γ*_*C*_ = *γ*_*D*_ = *γ*_*E*_ = 0.01	S0001	S0101	S1001
*ϕ*_*D*_ = 0.01, *ϕ*_*E*_ = 0.015, *γ*_*B*_ = *γ*_*C*_ = *γ*_*D*_ = *γ*_*E*_ = 0.01	S0010	S0110	S1010
*ϕ*_*D*_ = 0.01, *ϕ*_*E*_ = 0.01, *γ*_*B*_ = *γ*_*C*_ = *γ*_*D*_ = *γ*_*E*_ = 0.015	S0011	S0111	S1011

S00nn: Simulations are initialised with equal Democratic and Republican recruiting capacity and 1932 voting populations. S01nn: Simulations are initialised with slightly higher recruitment capacity for Republicans and 1932 voting populations. S10nn: Simulations are initialised with equal recruitment capacity for Democrats and Republicans and assuming same initial voting populations. From 2020 onwards: Snn00. Simulations with identical influence capacity of pro-Democrat and pro-Republican views outside the United States. Snn01. Simulations with a slight increase in pro-Democrat influence outside the United States. Snn10. Simulations with a slight increase in pro-Republican influence outside the United States. Snn11. Simulations with a slight increase in non-political influence outside the United States.

**Table 2:** Initial voting populations within the United States at *t*_0_ = 1932.

	Initial voting populations at *t*_0_ = 1932		
	S00nn	S01nn	S10nn
Democrats in US	22,821,277	22,821,277	19,291,265
Republicans in US	15,761,254	15,761,254	19,291,265
Non-partisan in US	34,650,000	34,650,000	34,650,000

Source data: US Census Bureau [45]. For agents outside the U.S., it is assumed that political tendencies are evenly split at the start of the simulation, with one-third non-partisan, one-third pro-Democrats, and one-third pro-Republicans. Simulations are initialised with an estimated population of 500 million people in N2, considered to have been directly or indirectly in potential contact with the US at that time. This figure is roughly equivalent to the population of Europe in 1932 [55].

**Fig 4:**
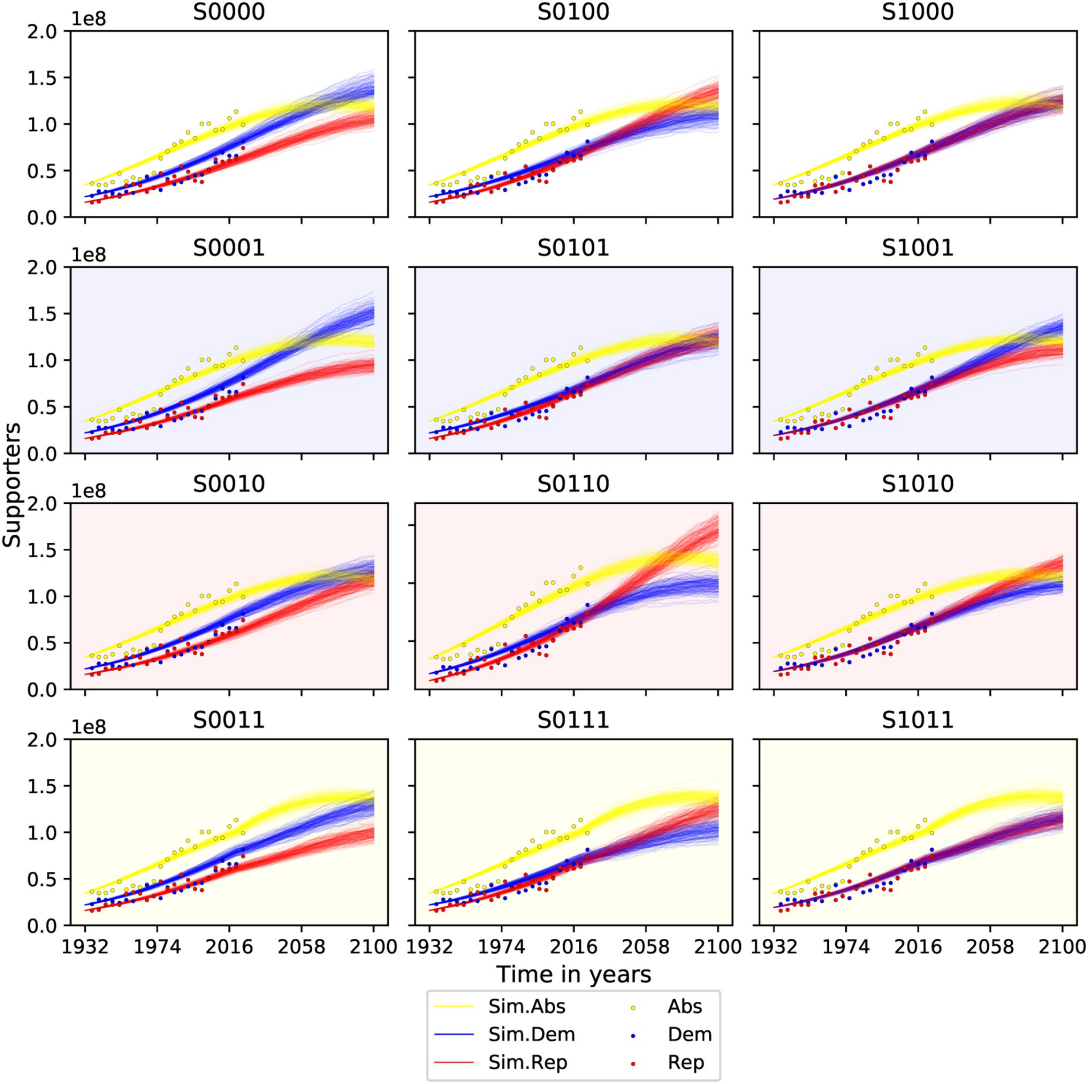
Supporters of each political group over time. The simulated scenarios correspond to the parameters described in Tables 1 and 2. Small shifts in political influence outside the country translate into changes in the domestic political map without the need for increases in domestic recruitment capacity. Voter turnout will grow at a faster rate than non-voters in the coming decades.

Our simulations (see Fig 4) show that if the Republicans’ recruiting capacity from Democrats increases by 0.005 per capita (in simulations S0100), they could reverse the balance of power by mid-21st century. However, assuming equal cross-party recruiting capacity (in simulations S0000), Democrats would continue expanding their lead. This is due to their larger popular vote base, which enables more political influence and results in higher recruitment rates. Democrats might have an edge in raw numbers in terms of party identification, this doesn’t always translate to consistent electoral success.

One of our most significant findings is that over time, more people will align with a political party than leave one. By the end of the century, in most examined scenarios, the number of Democrats and Republicans will be nearly equal to the non-partisan group. The decline in political disaffection is driven by the parties’ strong ability to attract non-partisans. The ability of both Democrats and Republicans to recruit non-partisans is greater than political disaffection over time. An increase of *γ*_*B*_, *γ*_*C*_, *γ*_*D*_, *γ*_*E*_ by 0.005 in overall political disaffection (as in S0011, S0111, S1011) would only delay convergence. But all other conclusions hold.

Cross-border influence is pivotal in our model. Small shifts in political views outside the US can have significant domestic repercussions. For instance, if pro-Democrat views, *ϕ*_*D*_, increase by 0.005 (like in scenarios S0001, S0101, S1001), it bolsters the Democrats, widening their lead. On the other hand, growing support for Republican views abroad (as in S0010, S0110, S1010) can help the Republicans bridge the gap even without greater domestic recruitment capacity (as in S0010).

### Model validation

In this section, we describe the process of validating the performance of our proposed model. We evaluate the standard model S00nn using various metrics to assess its effectiveness in capturing the underlying patterns of voting populations in U.S. presidential elections from 1932 to 2020.

The cross-validation performed is a variant of rolling cross-validation called “fixed window cross-validation”. For each political group (i.e. Non-partisan, Democrats, Republicans), two arrays were provided: “observed data” and “simulated data”. The “observed data” array represents the actual observed values, while the “simulated data” array contains the predicted values generated by the model, averaged over 10000 simulations.

Regarding the observed and simulated data sets, the evaluation was performed using five fixed-window validation splits. The observed and simulated data arrays were divided into pairs of validation windows. Simulated data was tested against observed data in a process that was repeated five times, shifting the validation window forward in five overlapping pairs that covered the entire dataset. We repeated the cross-validation process *k* = 5 times, with each of the *k* validation windows used exactly once as the test set.

Each testing set corresponds to a subset of the available data that was used to evaluate the performance of the model. For each fold, the accuracy was calculated by comparing the predicted values (“test predicted”) to the observed values (“test observed”). The accuracy was measured as the relative error between the two, expressed as a percentage. We also computed the average accuracy: after calculating the accuracy for each fold, the average accuracy was computed by taking the mean of the accuracy values across all folds. We used the Mean Absolute Percentage Error (MAPE) to measure forecast accuracy. The average accuracy represents an overall measure of the model’s performance across the five test folds. It is the average of the percentage errors.

We compared model performance against the accuracy of a null model. To calculate the accuracy of the null model, we used a common baseline approach that consists of predicting the mean value of the observed data for all test instances.

The purpose of this cross-validation is to evaluate the model’s predictive performance on different segments of the observed data. By using fixed window splits, it allows for assessing the model’s ability to generalize across time and make accurate predictions for unseen data points.

Table 3 summarizes the validation results of our model. The model achieved an acceptable accuracy, with a MAPE of less than 15%. While this percentage error might seem relatively high, it is important to consider the context of predicting voting patterns over such a long time period. Voting behavior is influenced by numerous complex factors, and capturing the underlying trends accurately is challenging. Therefore, achieving a 15% MAPE or less can be considered a promising outcome.

**Table 3:** Cross-validation.

	Fixed window cross-validation					
	S00nn Dem	Null Dem	S00nn Rep	Null Rep	S00nn Abs	Null Abs
Fold 1	20.59	46.90	15.48	49.51	15.84	44.60
Fold 2	14.24	37.44	10.93	51.09	12.45	46.00
Fold 3	13.41	35.49	8.55	45.41	12.61	52.23
Fold 4	12.89	36.87	18.26	33.88	13.39	40.60
Fold 5	11.53	15.98	16.27	22.47	11.31	36.56
MAPE	14.53	34.53	13.90	43.53	13.21	44.00
SD	10.94	10.12	9.97	10.83	9.39	5.27
*R* ^2^	0.81	0.00	0.81	0.00	0.80	0.00

Percentage errors.

The model’s coefficient of determination (*R*^2^) was 0.81 for Democrats, 0.81 for Republicans and 0.80 for Non-partisan. This indicates that at least 80% of the observed variance in the voting patterns can be explained by the predicted patterns. This suggests that the model is successful in capturing a substantial portion of the overall trends and patterns in the voting behavior.

### Political significance

The selection of the United States (US) as a region of interest for model testing and validation is based on several justifications:

- Global political significance: The US holds a prominent position in global politics and has a significant influence on international affairs. As one of the world’s largest democracies and an economic and political powerhouse, studying the US’s electoral behavior is of interest to researchers and policymakers worldwide [56].
- Data availability and reliability: The availability of extensive and reliable data from the US Census Bureau, covering voting populations and election results from 1932 to 2020, provides a robust foundation for model validation [45]. Such comprehensive and longitudinal data is often not readily available for many other countries.
- Two-party electoral system: The US political system, with its two-party dominance, presents an electoral landscape that facilitates model comparisons. Analyzing the US electoral system using our dynamical two-party system allows for the exploration of various factors affecting voting patterns, such as recruitment capacity and cross-border political influences [57].
- Applicability: Studying the US in comparison to populations outside the country is useful for exploring the dynamics of democracy and voting patterns. The model can potentially be applied to other countries with similar political structures and characteristics [58, 59].
- Cross-border influence: Investigating the interactions between the US and other influential populations outside the US enables an assessment of cross-border political influence. Understanding how political ideas and behaviors in one country can impact another is crucial in our globally interconnected world [60].
- Policy implications: The US’s position as a major political player has implications for global policy decisions. Understanding the behaviour of the US electorate and its potential to influence political dynamics in other countries can inform policymaking and international relations strategies [61–63].
- Academic interest: Given the prominent role of the US in shaping global political narratives, academic interest in understanding its voting patterns and their long-term trends is substantial. Our research can contribute to political science, sociology, and other social science disciplines, showcasing the United States’ political behavior in a global context [64].

While using data from the US as a region of interest for model validation and testing has its advantages, it also comes with certain limitations. For example, although insights gained from studying the US can be valuable, generalizing findings to other countries may not always be straightforward. Moreover, treating the population outside of the US as a single entity oversimplifies the diverse global political landscape and can introduce bias in the model’s predictions. Any model, by its nature, simplifies reality. Stochastic differential equations, while powerful, make certain assumptions about the stochastic nature of political processes that may not fully represent the complexity of real-world political behavior.

Despite all these limitations, the model we present here constitutes a pioneering attempt to apply non-linear dynamical modeling to real-world voting data. It represents a valuable starting point in our pursuit of mathematical insights into political behavior.

## Discussion

Our model simulates a system with fixed ideologies competing for supporters and exerting influence within and across borders. In the real world this idealised situation does not exist. Yet the evidence from studies of two-party political systems, as in the United States, shows that the relatively stable ideological nature of partisan views and preferences can be visible for decades [65–67]. Partisan views can also exhibit relative stability across borders. For example, prosocial or authoritarian preferences seem to behave as stable competing ideologies influencing processes such as the within-country democratisation of political systems [68]. Under these assumptions of relative stability, our model can inform how ideological alignment and the influence of political factions across national borders can affect intra-country political competition in an increasingly globalised world. In a context of increasing access to Internet, where the world is gradually achieving universal connectivity [69, 70], individuals are increasingly exposed to news and opinion from beyond national borders [71, 72]. This can facilitate information exchanges primarily among individuals with similar ideological preferences, increasing the likelihood of echo chamber formation (especially on political issues) whose effects transcend national borders [73], affecting the political equilibrium at the national level [74].

Voters and non-voters are influential forces determining the political dynamics within the country [2, 3, 47–53]. But in contrast to previous models, where ideological competition is examined within a single voting population, our model formalises how these effects transcend national borders affecting the national political equilibrium. Our model shows the extent to which subtle shifts in the influence of a minority opinion abroad can trigger substantial political change within the country, even while keeping domestic recruitment capacity intact (see scenarios V and VI in Fig 2). In this way, our model formalises ideas about minority influence that challenge the traditional assumption that majority influence determines ideological equilibrium [75, 76]. If majorities were all-powerful, there would never be political change.

In the specific case of voting patterns in the US presidential election, as shown in Fig 4, the best-fit model across all examined scenarios suggests that voter turnout will grow at a faster rate than non-voters in the coming decades. This prediction is based on the model’s expectation that both Democrats and Republicans will be more successful in recruiting non-partisans than the rate at which voters become politically disaffected. If this trend continues, by the end of the century, the number of voters for each party could surpass the number of non-voters in several of the scenarios examined.

A slight increase in the recruitment capacity of pro-Democratic political factions outside the country could increase the historical voting gap between the two parties if the Republican party is not able to compensate by increasing its influence at home. Conversely, an increase in support for Republicans outside the borders would allow them to close the gap and without the need for increased domestic recruitment capacity. Our findings are further supported by previous research that gives more weight to non-voters [47–53] and international influence [8–11, 77, 78]. A high number of increasingly active non-voters coupled with greater exposure to news and opinion across borders suggests that the ideology behind political change can no longer be analysed only in domestic terms. The trend towards increased non-voters politicisation explains the decline in abstention predicted by our model for the coming decades.

The generalizability of these results to understand cross-border political competition in other countries depends on whether there is alignment of political factions across national borders. For example, while global connectivity has enabled ideological alignments in much of the industrialised world, there are large geographic areas in underdeveloped countries where the reach of cross-border information is limited to low percentages of the population [69, 70]. However, our model is easily generalisable to countries with high connectivity and multi-party systems.

Analyzing long-term voting patterns offers significant academic value and provides insights into the progression of democratic processes and electoral behaviors. While predicting long-term voting trends is challenging, our model is able to capture a substantial amount of the variability in voting behaviour. This contributes to the broader body of knowledge in the field. Notably, our model consistently predicts an increase in voter turnout, suggesting that in the forthcoming decades, non-voters will decrease relative to the votes cast for the Democratic and Republican parties.

So what can we learn from our model of political influence? The example we have illustrated here shows that small acts promoting a minority idea can trigger aggregate processes that eventually culminate in ideological change at the global level. A flutter in a foreign country’s political influence represents a small change in the condition of the system, which cascades into large-scale alterations.

Our model works with spatially unstructured populations, but we know from previous studies that homogeneously and heterogeneously mixed populations can have different effects on the transmission of social information [79–83]. An extension of the system of Eq (1) incorporating such control is an avenue for future research that could stabilise the system at different fixed points.

## Appendix A

In accordance with the parameters, terms and assumptions described above, the governing differential equations of the model can be written as follows:

$$\begin{array}{c} \left\{ \begin{array}{cc} \frac{dA_{1}}{dt}= & \mu_{1}N_{1}-k_{B}p_{B}A_{1}\frac{B}{N_{1}}-\left(1-k_{B}p_{B}\right)k_{D}p_{D}A_{1}\frac{D}{N_{2}}-k_{C}p_{C}A_{1}\frac{C}{N_{1}}-\left(1-k_{C}p_{C}\right)k_{E}p_{E}A_{1}\frac{E}{N_{2}}\\ & -\mu_{A_{1}}A_{1}+\gamma_{B}B+\gamma_{C}C\\ \frac{dB}{dt}= & k_{B}p_{B}A_{1}\frac{B}{N_{1}}+\left(1-k_{B}p_{B}\right)k_{D}p_{D}A_{1}\frac{D}{N_{2}}-\phi_{C}B\frac{C}{N_{1}}-\left(1-\phi_{C}\right)\phi_{E}B\frac{E}{N_{2}}+\phi_{B}C\frac{B}{N_{1}}\\ & +\left(1-\phi_{B}\right)\phi_{D}C\frac{D}{N_{2}}-\mu_{B}B-\gamma_{B}B\\ \frac{dC}{dt}= & k_{C}p_{C}A_{1}\frac{C}{N_{1}}+\left(1-k_{C}p_{C}\right)k_{E}p_{E}A_{1}\frac{E}{N_{2}}-\phi_{B}C\frac{B}{N_{1}}-\left(1-\phi_{B}\right)\phi_{D}C\frac{D}{N_{2}}+\phi_{C}B\frac{C}{N_{1}}\\ & +\left(1-\phi_{C}\right)\phi_{E}B\frac{E}{N_{2}}-\mu_{C}C-\gamma_{C}C\\ \frac{dA_{2}}{dt}= & \mu_{2}N_{2}-k_{D}p_{D}A_{2}\frac{D}{N_{2}}-\left(1-k_{D}p_{D}\right)k_{B}p_{B}A_{2}\frac{B}{N_{1}}-k_{E}p_{E}A_{2}\frac{E}{N_{2}}-\left(1-k_{E}p_{E}\right)k_{C}p_{C}A_{2}\frac{C}{N_{1}}\\ & -\mu_{A_{2}}A_{2}+\gamma_{D}D+\gamma_{E}E\\ \frac{dD}{dt}= & k_{D}p_{D}A_{2}\frac{D}{N_{2}}+\left(1-k_{D}p_{D}\right)k_{B}p_{B}A_{2}\frac{B}{N_{1}}-\phi_{E}D\frac{E}{N_{2}}-\left(1-\phi_{E}\right)\phi_{C}D\frac{C}{N_{1}}+\phi_{D}E\frac{D}{N_{2}}\\ & +\left(1-\phi_{D}\right)\phi_{B}E\frac{B}{N_{1}}-\mu_{D}D-\gamma_{D}D\\ \frac{dE}{dt}= & k_{E}p_{E}A_{2}\frac{E}{N_{2}}+\left(1-k_{E}p_{E}\right)k_{C}p_{C}A_{2}\frac{C}{N_{1}}-\phi_{D}E\frac{D}{N_{2}}-\left(1-\phi_{D}\right)\phi_{B}E\frac{B}{N_{1}}+\phi_{E}D\frac{E}{N_{2}}\\ & +\left(1-\phi_{E}\right)\phi_{C}D\frac{C}{N_{1}}-\mu_{E}E-\gamma_{E}E \end{array}\right. \end{array}$$ (3)

where *N*_1_ = *A*_1_ + *B* + *C* being *A*_1_(0) > 0, *B*(0) ≥ 0, *C*(0) ≥ 0 and *N*_2_ = *A*_2_ + *D* + *E* being *A*_2_(0) > 0, *D*(0) ≥ 0, *E*(0)≥0. Adding the equations we see that *dN*_1_/*dt* = 0 and *dN*_2_/*dt* = 0.

Given that *k*_*B*_ stands for the ratio of members of *A*_1_ that are contacted by *B* per unit time, and that *p*_*B*_ is the probability of *B* convincing another agent per contact, then we have that the per capita recruitment rate of *B* from *A*_1_ is *β*_*B*_ = *p*_*B*_*k*_*B*_. The same applies to the rest of the equations, where: *β*_*C*_ = *p*_*C*_*k*_*C*_, *β*_*D*_ = *p*_*D*_*k*_*D*_, *β*_*E*_ = *p*_*E*_*k*_*E*_. Therefore, the model can be reduced to the following system:

$$\begin{array}{c} \left\{ \begin{array}{cc} \frac{dA_{1}}{dt}= & \mu_{1}N_{1}-\beta_{B}A_{1}\frac{B}{N_{1}}-\left(1-\beta_{B}\right)\beta_{D}A_{1}\frac{D}{N_{2}}-\beta_{C}A_{1}\frac{C}{N_{1}}-\left(1-\beta_{C}\right)\beta_{E}A_{1}\frac{E}{N_{2}}\\ & -\mu_{A_{1}}A_{1}+\gamma_{B}B+\gamma_{C}C\\ \frac{dB}{dt}= & \beta_{B}A_{1}\frac{B}{N_{1}}+\left(1-\beta_{B}\right)\beta_{D}A_{1}\frac{D}{N_{2}}-\phi_{C}B\frac{C}{N_{1}}-\left(1-\phi_{C}\right)\phi_{E}B\frac{E}{N_{2}}+\phi_{B}C\frac{B}{N_{1}}\\ & +\left(1-\phi_{B}\right)\phi_{D}C\frac{D}{N_{2}}-\mu_{B}B-\gamma_{B}B\\ \frac{dC}{dt}= & \beta_{C}A_{1}\frac{C}{N_{1}}+\left(1-\beta_{C}\right)\beta_{E}A_{1}\frac{E}{N_{2}}-\phi_{B}C\frac{B}{N_{1}}-\left(1-\phi_{B}\right)\phi_{D}C\frac{D}{N_{2}}+\phi_{C}B\frac{C}{N_{1}}\\ & +\left(1-\phi_{C}\right)\phi_{E}B\frac{E}{N_{2}}-\mu_{C}C-\gamma_{C}C\\ \frac{dA_{2}}{dt}= & \mu_{2}N_{2}-\beta_{D}A_{2}\frac{D}{N_{2}}-\left(1-\beta_{D}\right)\beta_{B}A_{2}\frac{B}{N_{1}}-\beta_{E}A_{2}\frac{E}{N_{2}}-\left(1-\beta_{E}\right)\beta_{C}A_{2}\frac{C}{N_{1}}\\ & -\mu_{A_{2}}A_{2}+\gamma_{D}D+\gamma_{E}E\\ \frac{dD}{dt}= & \beta_{D}A_{2}\frac{D}{N_{2}}+\left(1-\beta_{D}\right)\beta_{B}A_{2}\frac{B}{N_{1}}-\phi_{E}D\frac{E}{N_{2}}-\left(1-\phi_{E}\right)\phi_{C}D\frac{C}{N_{1}}+\phi_{D}E\frac{D}{N_{2}}\\ & +\left(1-\phi_{D}\right)\phi_{B}E\frac{B}{N_{1}}-\mu_{D}D-\gamma_{D}D\\ \frac{dE}{dt}= & \beta_{E}A_{2}\frac{E}{N_{2}}+\left(1-\beta_{E}\right)\beta_{C}A_{2}\frac{C}{N_{1}}-\phi_{D}E\frac{D}{N_{2}}-\left(1-\phi_{D}\right)\phi_{B}E\frac{B}{N_{1}}+\phi_{E}D\frac{E}{N_{2}}\\ & +\left(1-\phi_{E}\right)\phi_{C}D\frac{C}{N_{1}}-\mu_{E}E-\gamma_{E}E \end{array}\right. \end{array}$$ (4)

Now, because the transfer of agents between *B* and *C* due to their influence within the country results in a net amount of exchange, it follows that $\phi_{C}-\phi_{B}=\phi_{w_{1}}$. The same for *D* and *E*, where we have: $\phi_{E}-\phi_{D}=\phi_{w_{2}}$. Following the same reasoning, we also observe that there is a net transfer of agents due to cross-border influence, therefore $\left(1-\phi_{C}\right)\phi_{E}-\left(1-\phi_{B}\right)\phi_{D}=\phi_{b_{1}}$ and $\left(1-\phi_{E}\right)\phi_{C}-\left(1-\phi_{D}\right)\phi_{B}=\phi_{b_{2}}$. After this reduction, our system can be written as follows:

$$\begin{array}{c} \left\{ \begin{array}{cc} \frac{dA_{1}}{dt}= & \mu_{1}N_{1}-\beta_{B}A_{1}\frac{B}{N_{1}}-\left(1-\beta_{B}\right)\beta_{D}A_{1}\frac{D}{N_{2}}-\beta_{C}A_{1}\frac{C}{N_{1}}-\left(1-\beta_{C}\right)\beta_{E}A_{1}\frac{E}{N_{2}}\\ & -\mu_{A_{1}}A_{1}+\gamma_{B}B+\gamma_{C}C\\ \frac{dB}{dt}= & \beta_{B}A_{1}\frac{B}{N_{1}}+\left(1-\beta_{B}\right)\beta_{D}A_{1}\frac{D}{N_{2}}-\phi_{w_{1}}B\frac{C}{N_{1}}\\ & -\phi_{b_{1}}B\frac{E}{N_{2}}-\mu_{B}B-\gamma_{B}B\\ \frac{dC}{dt}= & \beta_{C}A_{1}\frac{C}{N_{1}}+\left(1-\beta_{C}\right)\beta_{E}A_{1}\frac{E}{N_{2}}+\phi_{w_{1}}B\frac{C}{N_{1}}\\ & +\phi_{b_{1}}B\frac{E}{N_{2}}-\mu_{C}C-\gamma_{C}C\\ \frac{dA_{2}}{dt}= & \mu_{2}N_{2}-\beta_{D}A_{2}\frac{D}{N_{2}}-\left(1-\beta_{D}\right)\beta_{B}A_{2}\frac{B}{N_{1}}-\beta_{E}A_{2}\frac{E}{N_{2}}-\left(1-\beta_{E}\right)\beta_{C}A_{2}\frac{C}{N_{1}}\\ & -\mu_{A_{2}}A_{2}+\gamma_{D}D+\gamma_{E}E\\ \frac{dD}{dt}= & \beta_{D}A_{2}\frac{D}{N_{2}}+\left(1-\beta_{D}\right)\beta_{B}A_{2}\frac{B}{N_{1}}-\phi_{w_{2}}D\frac{E}{N_{2}}\\ & -\phi_{b_{2}}D\frac{C}{N_{1}}-\mu_{D}D-\gamma_{D}D\\ \frac{dE}{dt}= & \beta_{E}A_{2}\frac{E}{N_{2}}+\left(1-\beta_{E}\right)\beta_{C}A_{2}\frac{C}{N_{1}}+\phi_{w_{2}}D\frac{E}{N_{2}}\\ & +\phi_{b_{2}}D\frac{C}{N_{1}}-\mu_{E}E-\gamma_{E}E \end{array}\right. \end{array}$$ (5)

We further reduce the system by defining the proportions with respect to the population of each country as *a*_1_ = *A*_1_/*N*_1_, *b* = *B*/*N*_1_, *c* = *C*/*N*_1_, *a*_2_ = *A*_2_/*N*_2_, *d* = *D*/*N*_2_, *e* = *E*/*N*_2_. After division we obtain the following differential equations:

$$\left\{\begin{array}{l} \frac{da_{1}}{dt}=\mu_{1}-\beta_{B}a_{1}b-(1-\beta_{B})\beta_{D}a_{1}d-\beta_{C}a_{1}c-(1-\beta_{C})\beta_{E}a_{1}e-\mu_{A_{1}}a_{1}+\gamma_{B}b+\gamma_{C}c\\ \frac{db}{dt}=\beta_{B}a_{1}b+(1-\beta_{B})\beta_{D}a_{1}d-\phi_{w_{1}}bc-\phi_{b_{1}}be-\mu_{B}b-\gamma_{B}b\\ \frac{dc}{dt}=\beta_{C}a_{1}c+(1-\beta_{C})\beta_{E}a_{1}e+\phi_{w_{1}}bc+\phi_{b_{1}}be-\mu_{C}c-\gamma_{C}c\\ \frac{da_{2}}{dt}=\mu_{2}-\beta_{D}a_{2}d-(1-\beta_{D})\beta_{B}a_{2}b-\beta_{E}a_{2}e-(1-\beta_{E})\beta_{C}a_{2}e-\mu_{A_{2}}a_{2}+\gamma_{D}d+\gamma_{E}e\\ \frac{dd}{dt}=\beta_{D}a_{2}d+(1-\beta_{D})\beta_{B}a_{2}b-\phi_{w_{2}}de-\phi_{b_{2}}dc-\mu_{D}d-\gamma_{D}d\\ \frac{de}{dt}=\beta_{E}a_{2}e+(1-\beta_{E})\beta_{C}a_{2}c+\phi_{w_{2}}de+\phi_{b_{2}}dc-\mu_{E}e-\gamma_{E}e \end{array}\right.$$ (6)

Now, let us denote the equilibrium of the above system as ($a_{1}^{*}$, b^*^, c^*^, $a_{2}^{*}$, d^*^, e^*^) and therefore $a_{1}^{*}$ = $A_{1}^{*}$/*N*_1_, b^*^ = B^*^/*N*_1_, c^*^ = C^*^/*N*_1_, $a_{2}^{*}$ = $A_{2}^{*}$/*N*_2_, d^*^ = D^*^/*N*_2_, e^*^ = E^*^/*N*_2_, where ($A_{1}^{*}$, B^*^, C^*^, $A_{2}^{*}$, D^*^, E^*^) represents the equilibrium of the unreduced system. Since the population of agents in each country remains constant as given by *N*_1_ = *A*_1_ + *B* + *C* and *N*_2_ = *A*_2_ + *D* + *E*, we deduce *a*_1_ + *b* + *c* = 1 and *a*_2_ + *d* + *e* = 1 for the reduced system. Using this fact, the reduced model system will be given by the following four differential equations:

$$\begin{array}{c} \left\{ \begin{array}{c} \frac{db}{dt}=\beta_{B}\left(1-b-c\right)b+\left(1-\beta_{B}\right)\beta_{D}\left(1-b-c\right)d-\phi_{w_{1}}bc-\phi_{b_{1}}be-\mu_{B}b-\gamma_{B}b\\ \frac{dc}{dt}=\beta_{C}\left(1-b-c\right)c+\left(1-\beta_{C}\right)\beta_{E}\left(1-b-c\right)e+\phi_{w_{1}}bc+\phi_{b_{1}}be-\mu_{C}c-\gamma_{C}c\\ \frac{dd}{dt}=\beta_{D}\left(1-d-e\right)d+\left(1-\beta_{D}\right)\beta_{B}\left(1-d-e\right)b-\phi_{w_{2}}de-\phi_{b_{2}}dc-\mu_{D}d-\gamma_{D}d\\ \frac{de}{dt}=\beta_{E}\left(1-d-e\right)e+\left(1-\beta_{E}\right)\beta_{C}\left(1-d-e\right)c+\phi_{w_{2}}de+\phi_{b_{2}}dc-\mu_{E}e-\gamma_{E}e \end{array}\right. \end{array}$$ (7)
